# Avaliação da Gravidade da Doença Arterial Coronariana em Pacientes Tratados com Quimioterapia: A Necessidade Adicional da Cardio-Oncologia

**DOI:** 10.36660/abc.20200408

**Published:** 2020-06-29

**Authors:** Matthew E. Harinstein

**Affiliations:** 1 Heart and Vascular Institute University of Pittsburgh Medical Center PittsburghPA EUA Heart and Vascular Institute, University of Pittsburgh Medical Center, Pittsburgh, PA- EUA

**Keywords:** Doença da Artéria Coronariana/quimioterapia, Neoplasias, Cardiotoxicidade, Taxa de Sobrevida, Infarto do Miocárdio/complicações, Trombose/complicações

A toxicidade cardiovascular relacionada às terapias contra o câncer é reconhecida há anos.^1^ O número e os tipos de toxicidade aumentaram rapidamente devido a vários fatores, incluindo terapias novas e aperfeiçoadas e regimes de tratamento que resultaram em maior sobrevida para os pacientes. Essa é a base para o crescente campo da cardio-oncologia, para ajudar a identificar cardiotoxicidade e cujo objetivo é minimizar os resultados adversos.

Estudos sobre cardiomiopatias relacionadas às antraciclinas, geralmente irreversíveis, e trastuzumabe, tipicamente reversíveis, bem como as cardiotoxicidades reconhecidas mais recentemente, incluindo miocardite relacionada a inibidores de *checkpoint* imune, fizeram a avaliação da doença cardíaca concomitante crucial no tratamento de pacientes em tratamento para câncer.^1 , 2^ A doença arterial coronariana (DAC) também é uma consequência de terapias contra o câncer e eventos coronarianos adversos, como infarto do miocárdio e trombose, podem complicar o tratamento e ter um desfecho desfavorável. Assim, uma maior compreensão dos efeitos adversos de terapias específicas é crucial para avaliar o estado clínico dos pacientes e tomar decisões sobre estratégias de tratamento, a fim de maximizar os resultados gerais, tanto oncológicos quanto cardíacos. A DAC tem sido associada à radioterapia,^3 , 4^ e o risco e a gravidade anatômica da DAC relacionados ao tratamento com radioterapia já foram descritos.^5 , 6^ Em um estudo com 152 sobreviventes de câncer torácico submetidos à radioterapia, os investigadores observaram que os pacientes do estudo tinham escores SYNTAX mais altos e corriam maior risco de desenvolver DAC anatomicamente grave, independente da quimioterapia.^6^ Embora se saiba que a DAC está presente em pacientes tratados com quimioterapia, independentemente da radioterapia, a associação entre a gravidade anatômica da DAC e a quimioterapia é menos conhecida.

Sabe-se que as síndromes coronárias agudas, incluindo trombose coronariana, infarto do miocárdio, angina e vasoespasmo, são complicações causadas por vários agentes quimioterápicos, o que afeta os resultados em curto e longo prazo.^7^ Os fatores de risco cardiovascular tradicionais, incluindo hipertensão, diabetes mellitus e tabagismo, estão presente em pacientes com câncer e tem sido sugerido que a DAC preexistente aumenta o risco de desenvolver DAC relacionada ao tratamento.^8^ Apesar da eficácia dos agentes quimioterápicos contra o câncer, os mecanismos potenciais que levam a eventos cardiovasculares indesejados incluem disfunção endotelial, agregação plaquetária, níveis reduzidos de óxido nitroso, níveis aumentados de espécies reativas de oxigênio e vasoespasmo.^9^ No entanto, o efeito que diferentes agentes quimioterápicos têm em relação à a gravidade anatômica e a complexidade da DAC podem ajudar ainda mais a estratificar os pacientes submetidos à quimioterapia, a fim de determinar quem pode estar em risco de eventos cardíacos adversos e / ou quem deve ter alterações no tratamento considerado.

Nesta edição dos Arquivos Brasileiros de Cardiologia, Yang et al.^10^ investigaram a associação entre quimioterapia e anormalidades anatômicas ateroscleróticas das artérias coronárias, com base na angiografia coronariana, em pacientes tratados por câncer de pulmão.^10^ O grupo de estudo transversal retrospectivo incluiu 94 pacientes, 36 dos quais foram submetidos à quimioterapia e os demais, não. Note-se que quase metade daqueles submetidos à quimioterapia também receberam radioterapia, enquanto apenas 7% daqueles que não foram submetidos à quimioterapia receberam radiação. Os autores descobriram que a gravidade da DAC, avaliada pelo escore SYNTAX, foi maior no grupo de quimioterapia comparado ao grupo de não-quimioterapia. Após análises univariadas e multivariadas, eles determinaram que a quimioterapia aumentou o risco de um escore SYNTAX alto e a quimioterapia aumentou o risco de DAC anatômica mais grave em 5,333 vezes.

Os pacientes da coorte exibiam fatores de risco tradicionais de DAC, incluindo idade avançada, hipertensão e tabagismo; entretanto, apenas metade fumava e aproximadamente 20% tinha diabetes mellitus. Não houve diferenças demográficas significativas entre os grupos de quimioterapia e não-quimioterapia. É importante ressaltar que os autores relataram os tipos de regimes de quimioterapia que os pacientes receberam. As quimioterapias à base de platina têm sido associadas a um risco em longo prazo 1,5 a 7 vezes maior de DAC e infarto do miocárdio; entretanto, a complexidade da DAC não está bem descrita.^7^ Na população estudada por Yang et al.,^10^ aproximadamente 78% dos pacientes receberam regimes de tratamento à base de platina. Eles observaram um risco ainda maior de DAC anatômica mais grave nesse grupo. Os autores concluem que a quimioterapia está associada à complexidade e gravidade anatômica da DAC e postulam que ela pode ser parcialmente responsável pelo maior risco de DAC em pacientes com câncer de pulmão. É importante observar que, embora o manejo médico deva ser a primeira estratégia de tratamento empregada no tratamento da DAC, as terapias invasivas não são proibitivas, apesar da presença de várias comorbidades.

Apesar da presença de coagulopatias e trombocitopenia, as quais podem estar presentes em pacientes que recebem quimioterapia, as mesmas não devem ser consideradas contraindicações para terapias coronárias invasivas. Demonstrou-se que a intervenção coronária percutânea (ICP) pode ser realizada com segurança em pacientes com contagem de plaquetas superior a 30.000 /mL após o acesso por micropuntura e realização de cuidadosa hemostasia.^11^ Desse modo, em pacientes com DAC obstrutiva que apresentam falha da terapia médica, uma estratégia de tratamento com ICP com colocação de stent farmacológico, com o menor tempo necessário de terapia antiplaquetária dupla, ainda deve ser considerada.^12^

As limitações do estudo são adequadamente descritas pelos pesquisadores. A amostra era pequena, e este foi um estudo retrospectivo de centro único, realizado em uma população específica de pacientes que tinha câncer de pulmão e foram submetidos à angiografia coronariana por suspeita de DAC. Um número menor de pacientes recebeu radioterapia no grupo não-quimioterápico. Metade dos pacientes no grupo de quimioterapia também recebeu radioterapia, potencialmente amplificando o efeito nas artérias coronárias. Como observado, seria útil saber o estágio do câncer de pulmão na apresentação inicial, uma vez que aqueles que receberam quimioterapia poderiam ter uma doença mais avançada e, consequentemente, mais inflamação por um período de tempo mais longo, o que pode promover a aterosclerose e contribuir para os resultados observados. Além disso, a correlação entre a gravidade anatômica da DAC e os eventos cardiovasculares clínicos em longo prazo não foi avaliada. A futura avaliação dos desfechos é importante para determinar se a presença de DAC mais complexa prediz um pior prognóstico nesse grupo de pacientes. Assim, entender não apenas a associação, mas também o efeito da quimioterapia na gravidade anatômica da DAC é importante ao planejar e monitorar a estratégia de tratamento de um paciente.

Yang et al.^10^ deram o próximo passo para entender a importância da DAC em pacientes tratados com quimioterapia, ao avaliar a gravidade e a complexidade da DAC. Isso destaca a crescente necessidade do campo da cardio-oncologia de investigar os efeitos e desfechos cardiovasculares em pacientes que têm e são tratados por câncer.

Com o objetivo de minimizar os eventos cardíacos imprevistos, investigações adicionais sobre este tópico, avaliando as muitas classes de agentes quimioterápicos e diferentes tipos de câncer são importantes para nossa compreensão de como tratar melhor os pacientes e prevenir eventos cardiovasculares adversos. O monitoramento dos resultados clínicos e a avaliação da DAC em futuros estudos clínicos prospectivos são necessários para validar o efeito da quimioterapia na gravidade anatômica e nos mecanismos subjacentes da DAC em pacientes tratados por câncer.
