# Supplementary material for: Avaliação da Gravidade da Doença Arterial Coronariana em Pacientes Tratados com Quimioterapia: A Necessidade Adicional da Cardio-Oncologia
Source: Arq Bras Cardiol. 2020 Jun 29;114(6):1013–4. [Article in Portuguese] doi: 10.36660/abc.20200408 (PMC8416125; doi:10.36660/abc.20200408)
Supplement: Translation

# Translation: Evaluating the Severity of Coronary Artery Disease in Patients Treated with Chemotherapy: The Further Need for Cardio-Oncology

**Authors:** Matthew E. Harinstein

**Affiliations:** 1 USA Heart and Vascular Institute, University of Pittsburgh Medical Center, Pittsburgh, PA -USA

**Keywords:** Coronary Artery Disease/radiotherapy, Neoplasms, Cardiotoxicity, Survival Rate, Myocardial Infarction/complications, Thromboses/complications

Cardiovascular toxicity related to cancer therapies has been recognized for years.^1^ The number and types of toxicities have increased rapidly due to several factors including new and improved therapies and treatment regimens which have resulted in patients living longer. This is the basis for the burgeoning field of cardio-oncology to help identify cardiotoxicities and aim to minimize adverse outcomes.

Cardiomyopathies related to anthracyclines, which are typically irreversible, and trastuzumab, typically reversible, as well as more recently recognized cardiotoxicities including myocarditis related to immune checkpoint inhibitors have made the evaluation of concomitant cardiac disease critical in the care of patients being treated for cancer.^1 , 2^ Coronary artery disease (CAD) is also a consequence of cancer therapies and adverse coronary events such as myocardial infarction and thrombosis can complicate treatment and result in poor outcomes. Thus, further understanding of the adverse effects of specific therapies is crucial to assessing patients’ clinical statuses and making decisions on treatment strategies in order to maximize overall outcomes, both oncologic and cardiac. CAD and has been associated with radiation therapy.^3 , 4^ The risk and anatomic severity of CAD related to radiotherapy treatment has been described.^5 , 6^ In a study of 152 thoracic cancer survivors who underwent radiotherapy, the investigators observed that the study patients had higher SYNTAX scores and were at a higher risk of developing anatomically severe CAD, independent of chemotherapy.^6^ While although CAD is known to be present in patients being treated with chemotherapy, independent of radiotherapy, the association between anatomic severity of CAD and chemotherapy is less well known.

Acute coronary syndromes, including coronary thrombosis, myocardial infarction, angina as well as vasospasm are known to be complications of several chemotherapy agents, which affects both short and long-term outcomes.^7^ Traditional cardiovascular risk factors including hypertension, diabetes mellitus and tobacco abuse are present in patients with cancer and it has been suggested that pre-existing CAD increases the risk of developing treatment-related CAD.^8^ Despite the effectiveness of chemotherapeutic agents against cancer, potential mechanisms leading to unintended cardiovascular events include endothelial dysfunction, platelet aggregation, reduced nitrous oxide levels, increased levels of reactive oxygen species and vasospasm.^9^ However, the effect that different chemotherapy agents have in regards to the anatomic severity and complexity of CAD may further help to risk stratify patients undergoing chemotherapy in order to determine who may be at risk of adverse cardiac events and or who should have alterations in their treatment considered.

In this issue of *Arquivos Brasileiros de Cardiologia* , Yang et al.^10^ investigated the association between chemotherapy and atherosclerotic anatomic abnormalities of coronary arteries, based on coronary angiography, in patients treated for lung cancer.^10^ Their retrospective cross-sectional study group included 94 patients, 36 of whom received chemotherapy and the remaining did not. It should be noted that nearly half of those who received chemotherapy also had radiation therapy, whereas only 7% of those who did not have chemotherapy had radiation. The authors found that the severity of CAD, as assessed by the SYNTAX score, was higher in the chemotherapy group compared to the non-chemotherapy group. After univariate and multivariate analyses, they determined that chemotherapy increased the risk of a high SYNTAX score and chemotherapy increased the risk of more severe anatomical CAD by 5.323 times.

Patients in the cohort exhibited traditional CAD risk factors including older age, hypertension and tobacco abuse, however, only half smoked and approximately 20% had diabetes mellitus. There were no significant demographic differences between the chemotherapy and non-chemotherapy groups. Importantly, the authors reported the types of chemotherapy regimens the patients received. Platinum-based chemotherapies have been associated with up to a 1.5- to 7-fold higher long-term risk of CAD and myocardial infarction, however, the complexity of CAD is not well described.^7^ In the population studied by Yang et al.^10^ approximately 78% of patients were treated with platinum-based regimens. They observed an even greater risk of more severe anatomical CAD in this group. The authors conclude that chemotherapy is associated with anatomical complexity and severity of CAD and postulate that it might partly account for the higher risk of CAD among lung cancer patients. It is important to note that while although medical management should be the first treatment strategy employed for the treatment of CAD, invasive therapies are not prohibitive despite the presence of several comorbidities.

Despite the presence of coagulopathies and thrombocytopenia which may be present in patients who receive chemotherapy, these should not be considered contraindications for invasive coronary therapies. It has been demonstrated that percutaneous coronary intervention (PCI) can be performed safely in patients with platelet counts greater than 30,000/mL after micropuncture access and achievement of careful hemostasis.^11^ Thus, in patients with obstructive CAD, who fail medical therapy, a treatment strategy of PCI with drug-eluting stent placement with the least length of required dual antiplatelet therapy should still be considered.^12^

The limitations of the study are fairly described by the investigators. The sample was small, and this was a single-center retrospective study, performed among a specific population of patients, who had had lung cancer and underwent coronary angiography for suspicion of CAD. A lower number of patients received radiotherapy in the non-chemotherapy group. Half of the patients in the chemotherapy group also received radiation therapy, thus potentially amplifying the effect on the coronary arteries. As noted, it would be helpful to know the stage of lung cancer at initial presentation, since those who received chemotherapy could have had more advanced disease and, consequently, more inflammation for a longer period of time, which may promote atherosclerosis and contribute to the results observed. Additionally, the correlation between anatomical severity of CAD and long-term clinical cardiovascular events was not assessed. The future assessment of outcomes is important to determine if the presence of more complex CAD portends worse prognosis in this group of patients. Thus, understanding not only the association, but also the effect of chemotherapy on anatomical severity of CAD is important when both planning and monitoring a patient’s treatment strategy.

Yang et al.^10^ took the next step in understanding the significance of CAD in patients treated with chemotherapy by evaluating the severity and complexity of CAD. This highlights the growing need for the field of cardio-oncology to investigate the cardiovascular effects and outcomes in patients who have and are treated for cancer. In order to hopefully minimize unanticipated cardiac events, further investigations of this topic evaluating the many classes of chemotherapeutic agents and different types of cancer are important to our understanding of how best to treat patients and prevent adverse cardiovascular events. Monitoring of clinical outcomes and CAD assessment during future prospective clinical studies are necessary to validate the effect of chemotherapy on the anatomical severity and underlying mechanisms of CAD in patients treated for cancer.
